# Density‐Dependent Response of Alpine Meadows to Plateau Zokor Disturbance on the Tibetan Plateau: Nonlinear Relationship and Ecological Thresholds

**DOI:** 10.1002/ece3.74561

**Published:** 2026-11-05

**Authors:** Yifan Zhao, Jing Yang, Shien Ren, Dongni Guo, Jin Li, Shoushu Pang, Lufan Yang, Tongxi Zhang, Hongyu Wang, Xiaoning Nan

**Affiliations:** ^1^ Key Laboratory of National Forestry and Grassland Administration on Management of Western Forest Bio‐Disaster, College of Forestry Northwest A&F University Yangling Shaanxi China; ^2^ Institute of Bailongjiang Forestry Science of Gansu Province Lanzhou China

**Keywords:** alpine meadow, degradation, ecological threshold, nonlinear relationship, plateau zokor

## Abstract

The plateau zokor (*Eospalax baileyi*) is a keystone subterranean rodent species in the alpine meadows of the Qinghai‐Tibet Plateau, and its burrowing and foraging activities exert complex dual effects on grassland ecosystems. Quantifying the relationship between zokor bioturbation and vegetation, ground‐surface and community characteristics, and identifying the ecological management threshold are crucial for sustainable land management. This study selected 21 typical alpine meadow sites along a disturbance gradient on the eastern Qinghai‐Tibet Plateau. We used unmanned aerial vehicle remote‐sensing to extract new mound density as a proxy for relative disturbance intensity and integrated multiple ground‐surveyed indicators into a grassland degradation index, so as to evaluate the association between overall grassland condition and disturbance intensity and to identify the key ecological management threshold. The results showed that vegetation cover and mean height declined linearly and the proportion of bare ground increased linearly with increasing disturbance intensity; community composition exhibited an inverted U‐shaped pattern in species richness and a U‐shaped pattern in the proportion of poisonous plants, whereas aboveground biomass and the proportion of edible forage showed no significant relationship with relative disturbance intensity. Furthermore, a nonlinear relationship was detected between zokor disturbance and the grassland degradation index, and we identified 166 new mounds·ha^−1^ as the ecological management threshold for plateau zokors; above this threshold, the tendency of grassland condition toward degradation became more pronounced, with surface denudation and deterioration of community composition emerging as the primary constraints on overall grassland condition. This study provides a quantitative basis for shifting alpine meadow management from conventional “pest control” toward threshold‐based adaptive management, and offers a reference for identifying and regulating bioturbation thresholds in other vulnerable ecosystems.

## Graphical abstract

By quantifying the nonlinear response of alpine meadows to plateau zokor activity, we established a robust ecological safety threshold of 166 mounds·ha^−1^ for sustainable land management. These findings provide a quantitative basis for shifting from conventional “pest control” to adaptive management that maintains plateau zokor populations within limits that support both biodiversity and ecosystem stability.

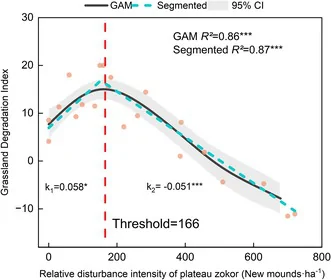

## 1 Introduction

The Qinghai‐Tibet Plateau serves as a critical ecological barrier and water conservation area in China (Wang, Liu, et al. 2022). Among its diverse landscapes, alpine meadows represent one of the most characteristic ecosystems, playing a pivotal role in maintaining regional ecological balance, regulating climate, conserving biodiversity, and supporting development of animal husbandry (Wang, Jia, et al. 2022). In recent years, however, alpine meadows have experienced increasingly severe degradation due to the combined pressures of global climate change and multiple ecological factors, including infrastructure construction, overgrazing, and rodent damage. This has led to a regressive shift in the structure and function of grassland ecosystems, characterized by declining productivity, reduced vegetation cover, and biodiversity loss, thereby posing a serious threat to regional ecological security and livelihoods of local herders (Fan et al. 2009; Zhang, Kang, et al. 2022).

Compared with other stressors, rodent outbreaks often exhibit more pronounced physical disturbance characteristics. Through activities such as burrowing, mound‐building, and foraging, rodents cause continuous disturbance to surface vegetation and soil structure. This accelerates grassland degradation and also drives the transition of grasslands from a generally degraded state toward a “rodent‐induced degradation” stage dominated by rodent activity, which can further evolve into typical “Heitutan” degradation (Xu et al. 2023). During this process, soil structure is disrupted, surface stability declines, and the ecosystem's original steady state is progressively undermined, leading to significantly lower self‐recovery capacity (Dong et al. 2013). Therefore, in‐depth understanding of the relationship between rodent infestation and grassland degradation is critical for effective formulation and implementation of grassland restoration strategies (Kang et al. 2007).

The plateau zokor (*Eospalax baileyi*) is a subterranean burrowing rodent endemic to the Qinghai‐Tibet Plateau, and a keystone species in alpine meadow ecosystems (Zhang et al. 2003). It spends most of its life underground, prefers moist and loose soils (Zhu, Tu, et al. 2025), and feeds mainly on belowground plant organs (Zhang and Liu 2003). Through burrowing, root foraging, soil translocation and mound building, it acts as a subterranean ecosystem engineer (Zhang et al. 2003). These activities continually modify plant communities and the distribution of materials and microhabitat conditions at the vegetation–soil interface, creating a spatial mosaic of mounds and inter‐mound vegetation (Bao et al. 2016; Niu, Yang, et al. 2019). Abandoned burrows can also provide nesting, breeding and refuge habitats for other grassland animals, especially invertebrates (Ye et al. 2022); the heterogeneous microhabitats formed by mounds and tunnels may further offer suitable conditions for seed germination and the regeneration of belowground bud banks (Xiang et al. 2021). The plateau zokor therefore plays a crucial role in sustaining food webs and in driving energy flow and nutrient cycling (Davidson et al. 2012).

This species plays a dual ecological role. At moderate population densities, it acts as a crucial facilitator and maintainer of ecosystem function. Its burrowing activities enhance soil aeration, accelerates nutrient cycling and increases soil heterogeneity, and promotes plant colonization and drives plant community succession (Ye et al. 2022). In contrast, when its population grows out of control, mound density rises accordingly, and the resulting ecological effects become increasingly negative; in particular, excessive feeding on roots severely damages vegetation structure (Hu et al. 2017), and frequent burrowing and mound‐building transport substantial volumes of deep soil to the surface, forming dense mounds that cover vegetation and reduce effective grazing area (Wang et al. 1993). In extreme cases, these processes reduce plant height, cover and above‐ and belowground biomass, suppress plant diversity, and cut forage yield and grassland productivity, culminating in severe degradation, widespread vegetation mortality and bare patches where the soil is completely exposed, posing a serious threat to the local environment and to the sustainability of pastoralism (Xie et al. 2013; Zhu, Tu, et al. 2025). Given the complex ecological role of this species, management strategies must balance its ecological benefits with the need for damage control. Maintaining disturbance intensity within a reasonable range of dynamic equilibrium allows the grassland to reap the zokor's benefits while limiting its destructive impacts, and this is essential for safeguarding the health and long‐term sustainability of alpine grassland ecosystems.

In pest management, the economic threshold is defined as the pest population density at which the cost of control equals the benefits gained, with a focus on maximizing economic returns (Foresti et al. 2017; Wolff 2022); in contrast, the ecological threshold refers to the population density at which pest activity significantly impairs ecosystem functions and services, emphasizing the maintenance of ecosystem health and stability (Andersen et al. 2009; Hua et al. 2024). While economic thresholds are applicable in scenarios focused on economic production, they exhibit inherent limitations when management objectives involve complex ecosystem services and system stability (Steinmann et al. 2021). In pest control, developing a comprehensive monitoring and prevention system to maintain pest density below the damage‐inducing threshold is a critical measure for ensuring the healthy succession and long‐term stability of natural grassland ecosystems (Chen and Li 2025). However, there has been little research on how to effectively integrate pest control thresholds into grassland degradation and restoration management from an ecological benefit perspective (Peterson et al. 2010). For grasslands that serve both as ecological barriers and production bases, current pest management standards are primarily based on economic thresholds, and the lack of systematic consideration of ecological thresholds makes it difficult to provide a balance between pest control and ecological protection (Hua 2025). Determining the ecological management threshold for plateau zokor disturbance in alpine meadow ecosystems is therefore essential; identifying the disturbance level at which grassland conditions begin to undergo sustained adverse changes could provide a basis for proactive monitoring and adaptive management.

Against this background, we conducted a field survey along a natural gradient of plateau zokor disturbance in alpine meadows on the eastern Qinghai‐Tibet Plateau. We focused on two questions: (1) how different grassland ecological indicators vary along the zokor disturbance gradient; and (2) how relative zokor disturbance intensity is associated with overall grassland condition. On this basis, a management‐relevant ecological threshold was identified. Our findings deepen the understanding of the ecological linkages between plateau zokor disturbance and the vegetation, ground‐surface conditions, community composition, and forage production of alpine meadows; provide a reference for grassland ecological research, resource conservation, and land management; and promote the integration of sustainable grassland management and integrated rodent pest management.

## 2 Materials and Methods

### 2.1 Study Site and Field Sampling

#### 2.1.1 Study Site

The study area is located at the northeastern margin of the Qinghai‐Tibet Plateau, at the junction of eastern Hezuo City and Zhuoni County (Figure 1). The terrain is relatively flat, with an average elevation of 3000–3500 m. The region is characterized by a mean annual temperature of approximately −0.5°C and an average annual precipitation of approximately 550–558 mm, primarily concentrated in summer. There is no absolute frost‐free period. The soil is predominantly subalpine meadow soil, and the vegetation type is alpine meadow, characterized by high vegetation cover and significant species diversity. The dominant plant species in this region include *Elymus nutans*, *Carex capillifolia*, *Poa pratensis* , *Bistorta vivipara* , *Argentina anserina* , and *Dasiphora fruticosa* . Common poisonous plant species include *Astragalus membranaceus*, *Pedicularis kansuensis*, and *Ranunculus tanguticus*.

**FIGURE 1:**
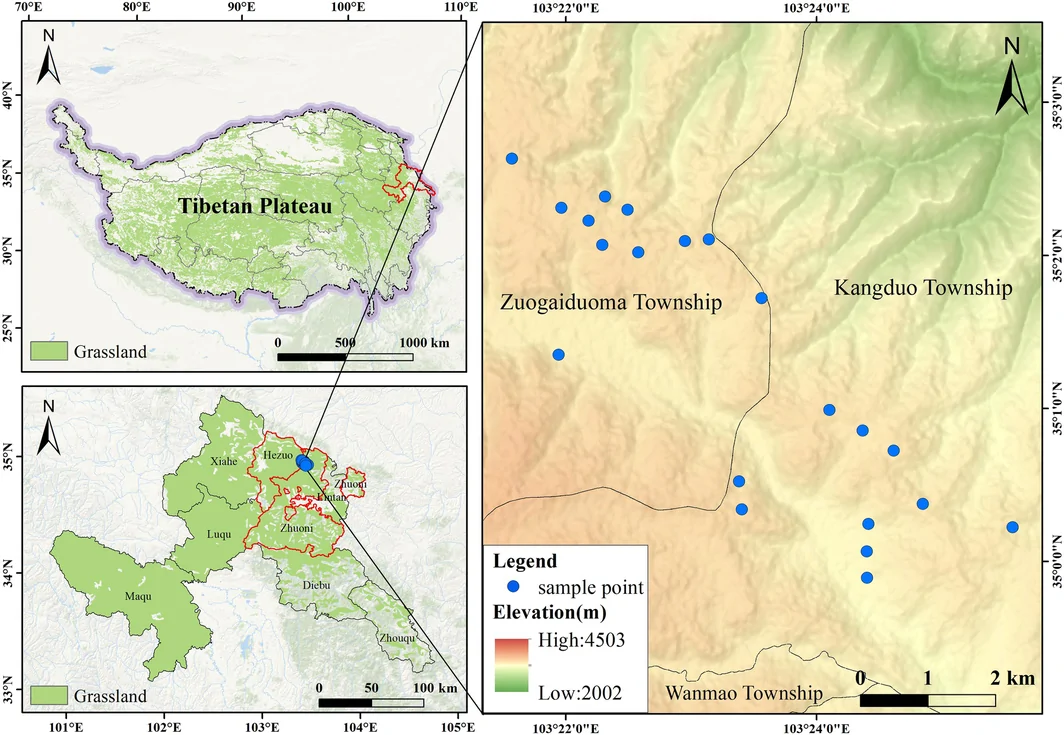
Map of the 21 survey sites on the northeastern Qinghai‐Tibet Plateau.

#### 2.1.2 Plot Selection and Mound Sampling

Interviews with local herders confirmed that the study area is managed under a seasonal grazing system comprising warm‐season and cold‐season pastures. All surveyed plots were located in alpine meadows designated as cold‐season pasture. These meadows were grazed mainly by yaks and Tibetan sheep, with approximately 1–1.2 head of livestock per hectare of grassland, and relatively stable grazing pressure. In May 2024, during the peak activity period of the plateau zokor, we selected 21 plots with relatively gentle slopes, consistent aspects and different plateau zokor disturbance intensities; each plot measured 100 m × 100 m, the mean distance between plots was approximately 500 m, and the mean slope ranged from 1.20° to 9.86°. For each plot, the relative disturbance intensity of plateau zokors was expressed as the number of newly formed mounds per unit area. We recorded newly formed mounds, which were characterized by a lack of vegetation on the surface (or only sparse plants at the edges), relatively high mound height, and surface cracking (Yang et al. 2020). Simultaneously, a DJI Mavic 2 Pro unmanned aerial vehicle (UAV), equipped with a 1‐in. CMOS image sensor, was deployed for low‐altitude aerial photography at a flight altitude of 25 m, covering a planned flight area of 120 m × 120 m. High‐resolution visible‐light images were obtained through image stitching using DJI Terra V4.4.6 software. The number of newly formed mounds of the plateau zokor was then determined through visual interpretation and validated using ground survey data. During field reconnaissance and ground verification, the plateau zokor was the only small subterranean herbivorous mammal with signs of activity in the surveyed plots.

#### 2.1.3 Vegetation Community Sampling

During July–August 2024 (the peak growing season), vegetation characteristic data were sampled across the 21 plots. Three 40‐m transects were established, radiating from the plot center toward the north, southeast, and southwest, with a 120° angle between adjacent transects. The point‐intercept method was used along each transect to survey the bare patch ratio and vegetation cover. Following the principles of representativeness and uniformity, three 1 m × 1 m quadrats were established within 5 m of either side of each transect in areas typical of the plot, totaling 63 quadrats across the 21 plots. Plant species within each quadrat were recorded, and species richness was calculated as the total number of species present in each quadrat. The mean vegetation height in each quadrat was calculated from 10 measurements of natural vegetation height. Aboveground biomass was determined by clipping all plants within the quadrats at ground level and weighing them. Edible forage was defined as all plant species excluding poisonous and unpalatable plants. The proportion of edible forage was calculated as the ratio of the number of edible forage species to the total number of plant species in the plot. Similarly, the proportion of poisonous plants was defined as the ratio of the number of poisonous plant species to the total number of plant species (Zhu, Fan, et al. 2025).

### 2.2 Development of a Grassland Degradation Assessment System

This study adopted an assessment framework oriented toward standardized grassland monitoring. Indicator selection balanced ecological significance, data availability, survey feasibility, and methodological consistency while ensuring comparability across regions and monitoring periods (Huang et al. 2024). On this basis, six measured indicators were selected as secondary indicators to reflect grassland health as represented by these four dimensions. Judgment matrices for the relative importance of the indicators were constructed through expert scoring, and weighting coefficients were calculated using the Analytic Hierarchy Process (AHP) to form the assessment system. Using the ideal reference system method, the observed value of each indicator in the assessment year was compared with its corresponding historical reference value within each dimension. Grassland resource data from the 1980s were used as the reference, and the reference value of each indicator is presented in (Table 1). Accordingly, the grassland health index (GHI) was constructed to assess grassland health as represented by the four dimensions in the study area. The GHI comprises four sub‐indices: the vegetation community status index (denoted as *V*), which characterizes vegetation growth; the land‐surface characteristic index (denoted as *L*), which describes surface conditions; the biodiversity status index (denoted as *B*), which characterizes biodiversity richness; and the forage production performance index (denoted as *F*), which reflects forage productivity and quality. The grassland degradation index (GDI) was further derived from the change in GHI between the assessment year and the baseline year to determine whether grassland degradation, as represented by the four dimensions, has occurred and to what extent; lower GDI values indicate poorer overall grassland condition (Table 2). The calculation formula for GDI is as follows:

(1) $$\mathrm{GHI}=\left(0.3\times V+0.2\times L+0.25\times B+0.25\times F\right)\times 100$$

**TABLE 1:** Reference values for grassland health indicators.

Primary indicator	Secondary indicators	Alpine meadow
1. Vegetation community characteristic	Vegetation cover (%)	90
2. Land surface characteristic	Proportion of bare ground (%)	10
3. Biodiversity	Species richness	10
4. Forage production performance	Aboveground biomass (kg·ha^−1^)	1000
	Proportion of edible forage (%)	85
	Proportion of poisonous plants (%)	10

**TABLE 2:** Classification of degradation levels in grasslands.

Serial number	Range of index variation	Grade
I	0 ≤ GDI	Not degenerated
II	−20 ≤ GDI < 0	Mild degeneration
III	−40 ≤ GDI < −20	Moderate degeneration
IV	GDI < −40	Severe degeneration

The formulas for the four sub‐indices were as follows:

(2) $$\begin{array}{c} V=\frac{\mathrm{VC}}{VC_{r}} \end{array}$$

(3) $$\begin{array}{c} L=\frac{BP_{r}}{\mathrm{BP}} \end{array}$$

(4) $$\begin{array}{c} B=\frac{\mathrm{SR}}{SR_{r}} \end{array}$$

(5) $$\begin{array}{c} F=\left(\frac{\mathrm{FP}}{FP_{r}}\times 0.5+\frac{\mathrm{EF}}{EF_{r}}\times 0.25+\frac{PH_{r}}{\mathrm{PH}}\times 0.25\right) \end{array}$$

(6) $$\begin{array}{c} \mathrm{GDI}=H_{a}-H_{r} \end{array}$$

where VC represents the vegetation cover (%), and $VC_{r}$ is its corresponding reference value; BP signifies the proportion of bare ground (%), with $BP_{r}$ serving as the reference; SR denotes the species richness, and $SR_{r}$ is the reference richness; FP indicates the fresh forage yield (kg·ha^−1^), while $FP_{r}$ is the reference yield; EF represents the proportion of edible forage (%), and $EF_{r}$ is the reference proportion; and PH indicates the proportion of poisonous plants (%), with $PH_{r}$ as the reference. Furthermore, GDI is the Grassland Degradation Index, while $H_{a}$ and $H_{r}$ represent the indices for the evaluation year and the baseline year, respectively. In this assessment, the grassland resource status from the 1980s was adopted as the baseline, with $H_{r}$ assigned a value of 80.

### 2.3 Statistical Analyzes

Data preprocessing and basic statistical analysis were performed using Microsoft Excel 2019. We analyzed the relationships between plateau zokor disturbance intensity and the grassland ecological indicators using linear and polynomial regression. Linear and nonlinear models [quadratic models and generalized additive models (GAMs)] were fitted to assess the relationship between plateau zokor relative disturbance intensity and grassland degradation index using the “mgcv” and “MASS” packages in R 4.2.2. The normality of the model residuals was assessed with the Shapiro–Wilk test, and all models satisfied the normality assumption. The optimal model was selected based on the Akaike Information Criterion (AIC) and Bayesian Information Criterion (BIC). To identify thresholds in the nonlinear relationship between plateau zokor relative disturbance intensity and grassland degradation index, the “segmented” and “chngpt” packages in R 4.2.2 were employed. Three threshold models were tested (“step,” “stegmented,” and “segmented”), and the best model was selected based on AIC, with its corresponding threshold value determined. A lower AIC value indicated a better model fit. Differences in AIC values of < 2 between two models were considered not significant. The step model represents the response variable as two constant fitted levels separated by a breakpoint, without estimating a continuous slope within either segment; the segmented model represents a continuous piecewise linear relationship, allowing different slopes before and after the breakpoint while maintaining continuity at the breakpoint; the stegmented model combines these features, allowing both a change in the response level at the breakpoint and different slopes on either side. We then avoided fitting a single complex function over the entire disturbance gradient, retaining all observations and describing the overall pattern with two piecewise linear relationships on either side of the breakpoint. Following threshold determination, Bayesian regression models were constructed using the “brms” package in R 4.2.2, with all variables standardized using Z‐scores, to examine the relative differences in the explanatory variables on either side of the threshold. Subsequently, Spearman correlation analysis was conducted using the ‘stats’ package to quantify the correlations between the relative disturbance intensity of plateau zokors and the six secondary indicators. All figures and tables were plotted using Origin 2025 software.

## 3 Results

### 3.1 Changes in Grassland Ecological Indicators Along a Plateau Zokor Disturbance Gradient

To investigate how grassland ecological indicators varied along the plateau zokor disturbance gradient, we examined the relationship between each indicator and relative disturbance intensity. As the relative disturbance intensity of plateau zokors increased, vegetation cover exhibited a significant linear decline (*R* ^2^ = 0.74, *p* = 3.4 × 10^−7^), while the proportion of bare patches showed a significant linear increase (*R* ^2^ = 0.39, *p* = 0.0015). Mean vegetation height also declined significantly and linearly with increasing disturbance intensity (*R* ^2^ = 0.24, *p* = 0.014). Species richness showed a significant quadratic relationship with relative disturbance intensity (*R* ^2^ = 0.23, *p* = 0.038). The regression curve was unimodal (inverted U‐shaped), suggesting that moderate zokor disturbance was beneficial for maintaining species diversity. The proportion of poisonous plants also exhibited a significant quadratic polynomial relationship (*R* ^2^ = 0.22, *p* = 0.040), displaying a U‐shaped trend, implying an increased risk of poisonous weed proliferation under high‐density disturbance. However, regression analysis revealed no significant relationship between the relative disturbance intensity and either aboveground biomass (*R* ^2^ = −0.050, *p* = 0.82) or proportion of edible forage (*R* ^2^ = 0.039, *p* = 0.19). Overall, the indicators differed in their responses to disturbance intensity (Figure 2).

**FIGURE 2:**
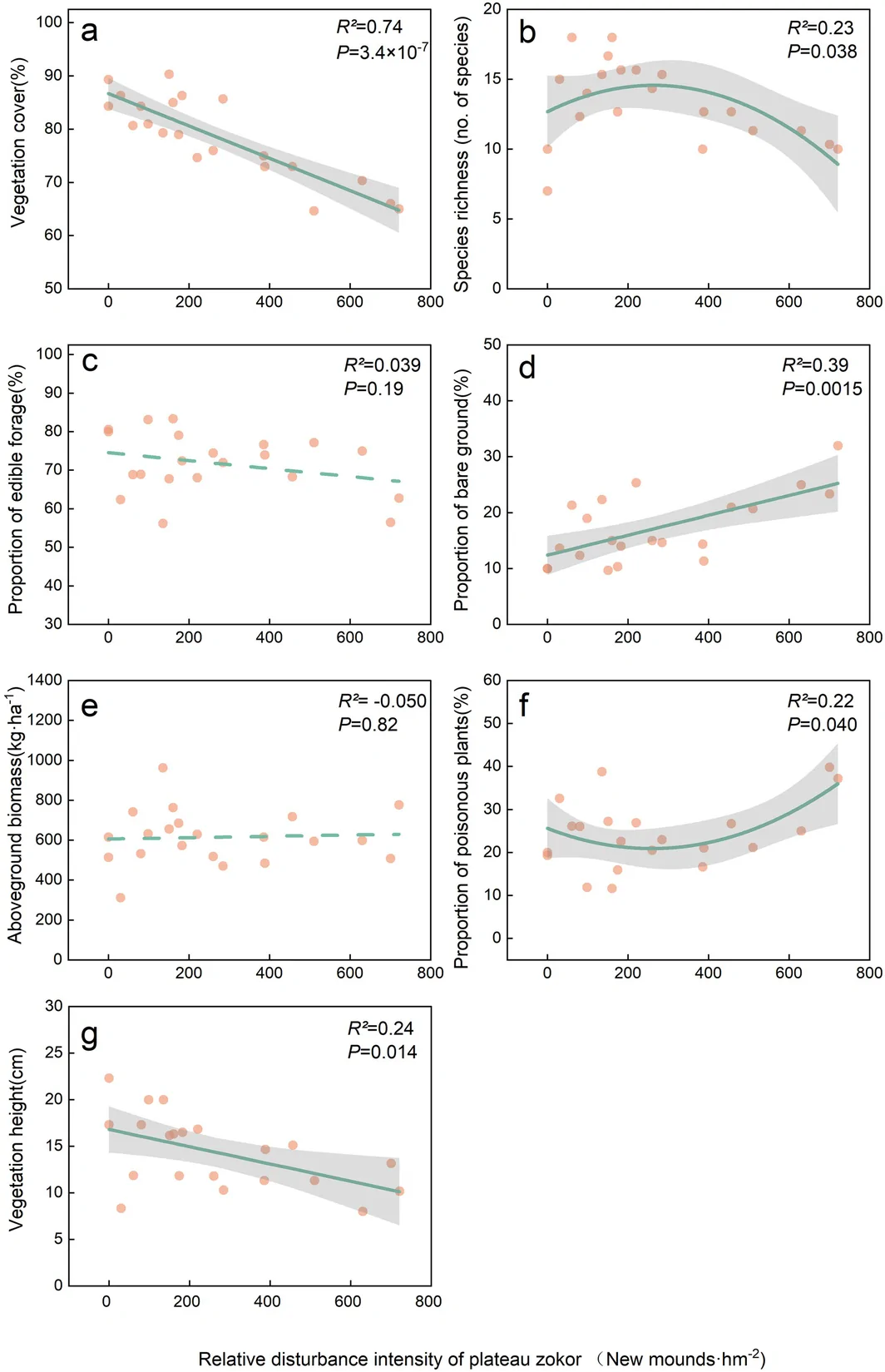
Relationships between grassland ecological indicators and plateau zokor new‐mound density: (a) vegetation cover, (b) Species richness, (c) Proportion of edible forage, (d) Proportion of bare ground, (e) Aboveground biomass, (f) Proportion of poisonous plants, and (g) Vegetation height.

### 3.2 Identification of Ecological Thresholds for Plateau Zokor Disturbance

#### 3.2.1 Nonlinear Relationship Between the Grassland Degradation Index and Plateau Zokor Disturbance

This study statistically tested the hypothesis of a nonlinear relationship between plateau zokors disturbance and the grassland degradation index by comparing linear, quadratic, and GAMs. Model performance results were summarized (Table 3). The GAM exhibited optimal fitting performance, with AIC and BIC values of 124.32 and 130.67, and high *R* ^2^ of 0.86 (*p* < 0.001). Compared with the linear model, the GAM showed substantial reductions in AIC and BIC, while *R* ^2^ increased from 0.64 to 0.86, a 34.37% improvement. Furthermore, compared to the quadratic model, it also had substantially lower AIC and BIC and a 10.25% higher *R* ^2^. These results indicated that the GAM better captured the complex dynamics between zokors and grassland degradation. The superior ability of the GAM to capture these complex nonlinearities led to the rejection of the linear relationship hypothesis, providing robust statistical evidence for a significant nonlinear association between plateau zokors and grassland degradation.

**TABLE 3:** Comparison of fitting performance among GAM, quadratic, and linear models.

Model	AIC	BIC	*R* ^2^	*p*	Conclusion
Linear	137.09	140.22	0.64	1.2 × 10^−5^	Reference model
Quadratic	128.94	133.12	0.78	1.1 × 10^−7^	Significantly outperforms linear models
GAM	124.32	130.67	0.86	1.9 × 10^−9^	Significantly outperforms linear and quadratic polynomial models

#### 3.2.2 Identification of the Ecological Management Threshold

To examine whether a threshold effect exists in the relationship between plateau zokor disturbance and changes in overall grassland condition, we compared three threshold models relating the number of new mounds to the grassland degradation index (GDI). All three models estimated a single breakpoint but differed in the form of change allowed at that breakpoint: the step model allowed an abrupt change in level with no change in slope; the segmented model described a continuous piecewise linear relationship, in which the slope changed at the breakpoint while the fitted values remained continuous; and the stegmented model allowed both a change in slope and a jump at the breakpoint. The AIC values for the segmented and stegmented models were 117.55 and 118.24, respectively. The difference between the two models was < 2, indicating no significant distinction, yet both were substantially lower than that of the step model (with differences > 2). Consequently, the final threshold for the plateau zokor was determined as the average of the two models (Table 4). The results showed that plateau zokor relative disturbance intensity had a significant threshold effect on changes in overall grassland condition. At the critical value of 166 new mounds·ha^−1^, GDI exhibited a directional shift: below this value, it increased with increasing disturbance, whereas above it, it showed a declining trend—possibly indicating that, beyond the threshold, grassland ecosystem resistance gradually declined and the tendency of grassland condition toward degradation became more pronounced (Figure 3).

**TABLE 4:** Ecological threshold identification model for plateau zokors.

Model	AIC	Threshold	Selection threshold
Segmented	117.55	150	166
Stegmented	118.24	182	
Step	135.33	388	

**FIGURE 3:**
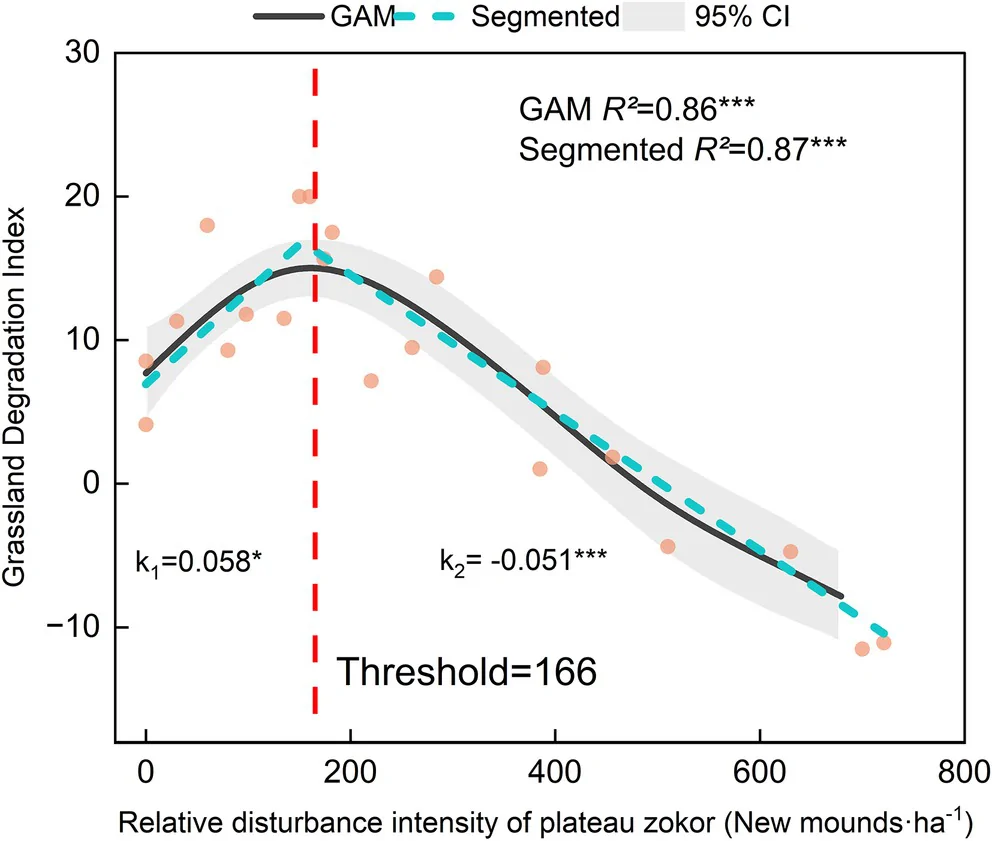
Relationship between plateau zokor new‐mound density and the grassland degradation index. The nonlinear trend is fitted using a generalized additive model (GAM), while the blue dashed lines represent the segmented regression on both sides of the threshold. Shaded areas indicate confidence intervals. The red dashed line indicates the identified ecological threshold for plateau zokors. *p < 0.05, ***p < 0.001.

### 3.3 Differences in Ecological Indicators Across the Threshold

To investigate how the roles of individual ecological indicators differed on either side of the threshold, we constructed Bayesian regression models and compared the posterior probability distributions of each indicator's effect across the threshold. At relatively low disturbance levels (*N* < 166 new mounds·ha^−1^), vegetation cover, plant species richness, and aboveground biomass had predominantly positive effects on the grassland degradation index (GDI), whereas the proportion of bare patches had a negative effect; the posterior probabilities of these effects were all high (*p* > 90%). In contrast, the proportions of poisonous plants and edible forage made no clear contribution to GDI (*p* < 90%). At relatively high disturbance levels (*N* > 166 new mounds·ha^−1^), vegetation cover, plant species richness, and aboveground biomass still had strong positive effects (*p* > 90%). However, the posterior probability of a negative effect of the proportion of bare patches reached 100%, and that of the proportion of poisonous plants rose markedly to 99.4%. These results indicate that, under high‐intensity disturbance, bare‐patch expansion and deterioration of community composition became the key factors limiting overall grassland condition. Beyond the threshold, physical habitat degradation and unfavorable changes in species composition began to dominate the declining trend in GDI (Figure 4).

**FIGURE 4:**
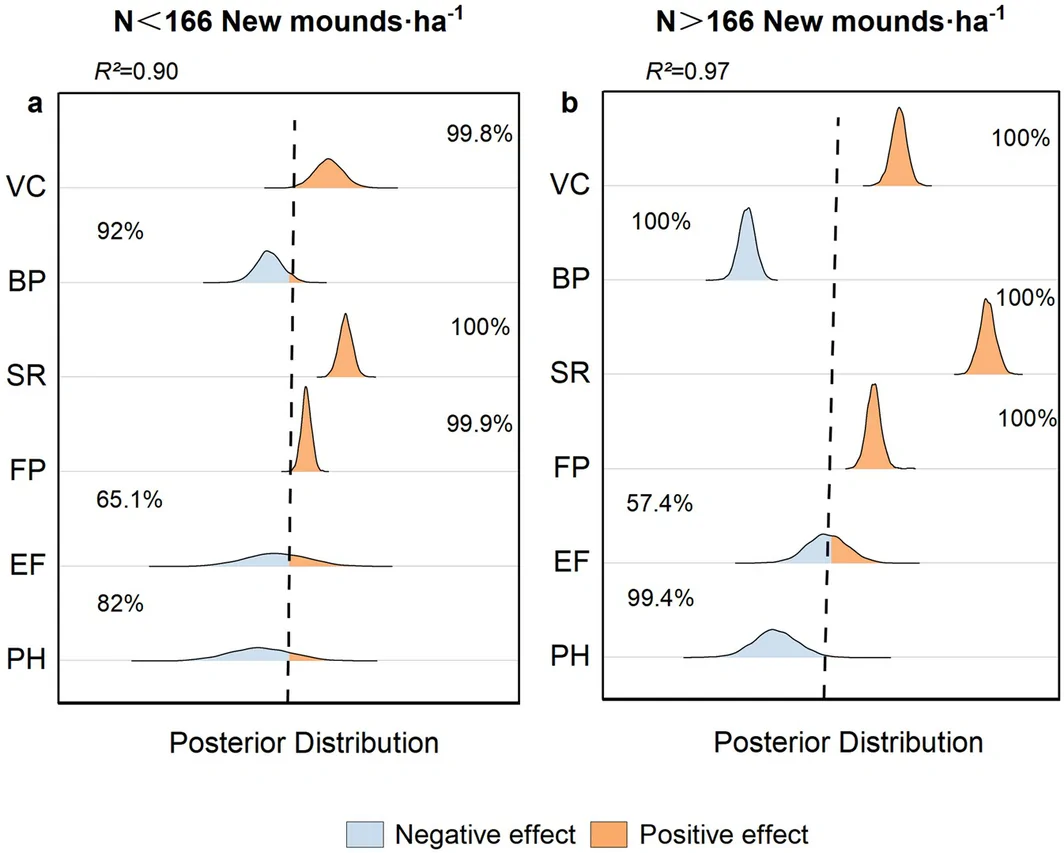
Bayesian posterior distributions of the effects of grassland ecological indicators on the grassland degradation index under (a) relatively low (< 166 new mounds·ha^−1^) and (b) relatively high (> 166 new mounds·ha^−1^) plateau zokor disturbance levels. The values represent the posterior probability (*p*) of an effect, with *p* > 90% considered significant. The explanatory power of the Bayesian model is expressed using Bayesian *R* ^2^, which effectively quantifies the uncertainty in model explanation. Variable codes: VC = vegetation cover (%); BP = proportion of bare patches (%); SR = species richness; FP = forage production (kg·ha‐1); EF = proportion of edible forage (%); PH = proportion of poisonous plants (%).

Spearman's correlation analysis revealed that at low relatively low disturbance levels (*N* < 166 new mounds·ha^−1^), new‐mound density was significantly positively correlated with species richness (*p* < 0.05) and with aboveground biomass (*p* < 0.05), but showed no significant correlations with vegetation cover, the proportion of bare patches, the proportion of edible forage, or the proportion of poisonous plants. At relatively high disturbance levels (*N* > 166 new mounds·ha^−1^), new‐mound density was significantly positively correlated with the proportion of bare patches (*p* < 0.05) and with the proportion of poisonous plants (*p* < 0.05), and significantly negatively correlated with vegetation cover (*p* < 0.001) and species richness (*p* < 0.01). No significant correlations were detected with aboveground biomass or the proportion of edible forage (Figure 5).

**FIGURE 5:**
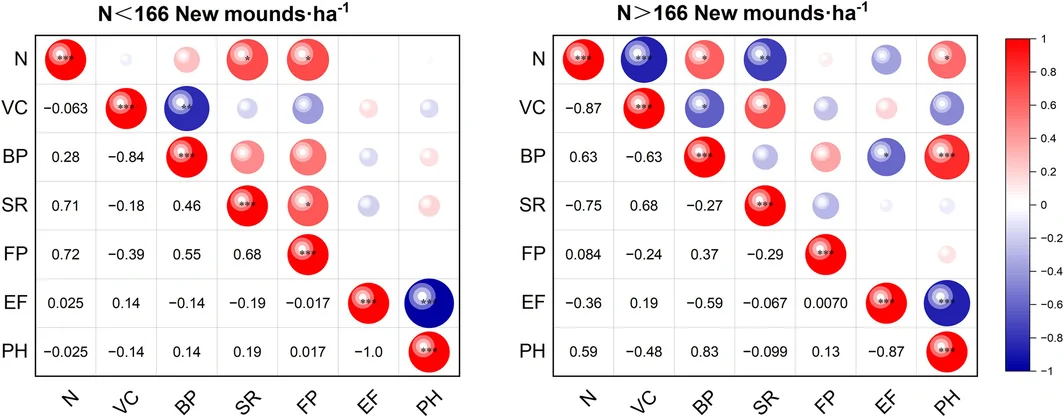
Heat map illustrating the Spearman's correlations between the number of new plateau zokor mounds and grassland ecological indicators. *p < 0.05, **p < 0.01, and ***p < 0.001. Variable codes: VC = vegetation cover (%); BP = proportion of bare patches (%); SR = species richness; FP = forage production (kg·ha−1); EF = proportion of edible forage (%); PH = proportion of poisonous plants (%).

## 4 Discussion

### 4.1 Responses of Grassland Ecological Indicators to the Disturbance of Plateau Zokors

The plateau zokor is a keystone subterranean rodent in alpine meadow ecosystems. Its activities directly alter surface features and soil structure, and also indirectly regulate resource allocation within plant communities by shaping microhabitats, thereby influencing grassland health (Zhang et al. 2003). Our results indicated that among various ecological indicators, vegetation cover, the proportion of bare patches, and mean vegetation height were the most sensitive to zokor disturbance, exhibiting significant linear relationships with relative disturbance intensity (Figure 2a,d,g). Mounding activity is the proximate driver of increased bare soil exposure; newly formed mounds disrupt the original vegetation and turf layers, leaving the soil vulnerable to wind and water erosion, which exacerbates degradation (Chen et al. 2021). Furthermore, vegetation recovery on mounds is protracted, taking several years to transition from pioneer annual weeds to the re‐establishment of high‐quality native forage (Zhang et al. 2023). If the rate of new mound formation consistently outpaces recovery, the system enters a maladaptive cycle in which destruction exceeds repair, leading to irreversible degradation (Xiang et al. 2009). Vegetation height is a non‐destructive structural indicator whose changes may reflect root damage caused by belowground foraging: plateau zokors feed mainly on the roots of perennial herbaceous plants (Zhang, Lin, et al. 2022); root damage at foraging tunnels exerts marked negative effects on plants (Zhang and Liu 2002); and damage to belowground plant parts is accompanied, to some extent, by reduced aboveground growth (Zvereva and Kozlov 2012). This suggests that incorporating non‐destructive structural indicators such as height into the monitoring of damage caused by subterranean rodents such as the plateau zokor may help capture the aboveground manifestation of belowground damage.

With regard to community composition, species richness followed a unimodal pattern along the plateau zokor disturbance gradient, the proportion of poisonous species in the total number of species followed a U‐shaped pattern, and no significant relationship was found for the proportion of edible forage (Figure 2b,c,f). These patterns are to some extent consistent with the intermediate disturbance hypothesis: moderate disturbance may improve soil aeration and nutrient availability through burrowing and mounding (Wang et al. 1993; Zhang and Liu 2003; Zhang et al. 2003), and, by burying vegetation and turning over soil, it disrupts the continuous aboveground distribution of dominant species and opens up space for the colonization and growth of companion and successional pioneer species (Niu, Yang, et al. 2019). In parallel, the preferential feeding of zokors on certain plant roots, particularly the taproots of dicotyledonous species, further reshapes competitive relationships among species within the community (Zhang and Liu 2003). The combined action of these processes increases species richness at low‐to‐intermediate disturbance levels; during this stage, the proportion of poisonous species declines, indicating that the net increase in non‐poisonous species outpaces that of poisonous species—even when the number of poisonous species also increases, its proportion is diluted by the faster increase in total species richness. As plateau zokor disturbance continues to intensify, the grassland enters a stage of degradation succession, in which soil properties and nutrient cycling may change, highly palatable forage declines markedly, and poisonous species become persistently established and expand rapidly, so that their proportion rises instead (Huang 2004).

In this study, we found no significant change in aboveground biomass along the plateau zokor disturbance gradient (Figure 2e), a discrepancy possibly reflecting a short‐term compensatory strategy in alpine meadows. Previous research suggests that zokor burrowing alters the vertical and horizontal distribution of soil nutrients, creating heterogeneity that may alleviate competition for resources and space among plants (Bao et al. 2016). In addition, compensatory growth at mound edges makes the productivity of the surrounding vegetation markedly higher than that in other areas (Li et al. 2023). Studies have shown that aboveground biomass does not differ significantly among zokor disturbance levels, whereas the importance values and biomass proportions of forbs increase with disturbance intensity, compensating for the decline of sedges and keeping total community biomass stable, while species diversity also increases with disturbance intensity (Chu et al. 2020). On the other hand, the plateau zokor is a typical subterranean herbivore that feeds mainly on plant roots and rhizomes (Cai et al. 2022), so its damage acts first on belowground parts. During the degradation of alpine meadows, belowground biomass may begin to decline at an early stage of disturbance, whereas the response of aboveground biomass lags behind (Peng et al. 2019); marked aboveground losses may become apparent only when degradation worsens and disturbance intensity increases further. On the one hand, roots and rhizomes are the direct recipients of disturbance, and belowground biomass reflects the disturbance process earlier and more sensitively than aboveground biomass (Peng et al. 2019). On the other hand, belowground biomass also constitutes the bulk of community biomass and carbon storage in alpine meadows, so neglecting the belowground component would make assessments based on aboveground indicators systematically over‐optimistic (Yang et al. 2009). Moreover, the plots comprise a mosaic of mounds of different ages and intermound vegetation, and differences among mounds of different ages in vegetation recovery and community composition may further increase the among‐plot variation in aboveground biomass (Wang et al. 2008).

A certain coupling relationship exists between plateau zokors and vegetation, through which zokors may affect plant communities directly or indirectly: burrowing and mounding act directly on the community by burying plants, destroying the sod, and altering microtopography, and act indirectly on vegetation by changing soil formation, aeration, and mixing, thereby creating nutrient heterogeneity between mounds and intermound areas (Bao et al. 2016; Zhang et al. 2003). The effects of both pathways are ultimately expressed largely in the vegetation layer, so evaluating the total effects of zokor disturbance on individual indicators is important. The multi‐indicator changes we observed provide evidence for the existence of this coupling relationship; as a key mediating variable, soil properties should be measured and studied simultaneously, which will help decompose total effects into direct and indirect pathways and thereby clarify the mechanisms by which plateau zokor damage drives grassland degradation.

### 4.2 Nonlinear Relationship Between Plateau Zokor Disturbance and Grassland Degradation Index

The impact of plateau zokor disturbance activities, such as burrowing and foraging, on alpine meadow ecosystems has long been debated, given their role as “ecosystem engineers” (Hua 2025). A key finding of this study is the empirical demonstration of a significant nonlinear relationship between zokor disturbance and grassland degradation index constructed in this study. Furthermore, a threshold effect was identified: once disturbance exceeds this level, the tendency of grassland condition toward degradation becomes more pronounced if managers do not intervene in time (Figure 3). The evolutionary process of ecosystems is often not a simple linear progression but a complex interplay of nonlinear feedbacks and regime shifts (Scheffer et al. 2001). Previous studies indicated that small burrowing mammals are intrinsic components of grassland ecosystems, playing a crucial role in maintaining grassland community dynamics and biodiversity (Li et al. 2023). Moderate zokor disturbance can have positive effects on plant communities, ecosystem balance, and nutrient cycling (Niu, Zhou, et al. 2019). However, driven by multiple factors, varying degrees of grassland degradation may create more suitable habitats for these animals, thereby promoting an increase in their population density and consequently intensifying their disturbance of grasslands (Cui et al. 2024). The continuous increase in zokor mounds significantly reduces grassland vegetation cover, with desirable forage species covered by mounds or dying from root herbivory (Song et al. 2023). Furthermore, extensive bare ground created by zokor mounds facilitates invasion and establishment of noxious weeds. Habitat destruction, leading to expansion of bare patches, exacerbates soil erosion, further inhibits vegetation recovery, and potentially accelerates the system's progression toward a degraded state (Yao et al. 2015). This transition may stem from the cumulative effects of high‐density zokor destructive behaviors surpassing their positive contributions, thereby imposing greater pressure on grassland ecosystems and leading to a decline in ecosystem resilience (Wang, Wan, et al. 2022). The effects of the ecological indicators changed on either side of the threshold, exhibiting asymmetry. In low‐disturbance intervals, the system possesses structural resilience (Harris 2010), allowing it to self‐repair through clonal growth (Liu et al. 2009). However, beyond the threshold, the coupling between zokor disturbance and negative indicators (bare patches and poisonous weeds) strengthens significantly. Frequent mounding induces the coalescence of bare patches, leading to the structural rupture of the turf layer and triggering a cascading effect of accelerated degradation (Zhang et al. 2003). In summary, this study provided empirical evidence for the nonlinear relationship and threshold effects of zokor disturbance in alpine meadows. Accordingly, alpine meadow management should maintain plateau zokor disturbance intensity below the ecological management threshold to curb further grassland degradation, while preserving the positive roles of this species in ecosystem functioning.

### 4.3 Limitations and Future Directions

In alpine meadows, plateau zokor disturbance does not act on vegetation in isolation but is embedded in a context shaped jointly by soil and grazing. This delimits the assessment boundary of this study and leaves room for future research. Owing to limited data availability, this study did not incorporate soil physicochemical properties into the assessment framework and lacked multi‐year historical data; the assessment therefore mainly reflects the vegetation and land‐surface dimensions and cannot yet fully capture the state of the entire ecosystem. The absence of belowground biomass from the assessment framework limits interpretation at the mechanistic level; future assessments of zokor disturbance should therefore monitor aboveground and belowground biomass simultaneously, together with biomass allocation among plant functional groups, so as to distinguish the apparent stability of total biomass from underlying shifts in community structure. In addition, livestock grazing may act in the same direction as zokor disturbance, and changes in grazing intensity may shift the identified threshold earlier or later along the disturbance gradient; although the plots shared a similar grazing background, the disturbance gradient was determined from mound coverage and spatial patterns at a single time point and therefore cannot reflect interannual fluctuations in disturbance intensity or historical legacy effects. Future research could incorporate soil physicochemical properties into monitoring and combine different grazing‐intensity treatments with long‐term continuous observations, so as to more completely capture changes in ecosystem state and further clarify the mechanisms by which zokor disturbance drives grassland degradation.

## 5 Conclusions

This study provides empirical evidence for the existence of a nonlinear relationship and a threshold effect between plateau zokor disturbance and changes in overall grassland condition in alpine meadows. The various grassland ecological indicators differed markedly in their patterns of change along the plateau zokor disturbance gradient. Vegetation cover, the proportion of bare patches, and mean vegetation height can serve as key indicators for monitoring the overall condition of grassland under plateau zokor disturbance, whereas aboveground biomass and the proportion of edible forage showed no clear patterns along the disturbance gradient. The relationship between plateau zokor disturbance and the grassland degradation index exhibited marked nonlinear threshold characteristics. This study identified 166 new mounds·ha^−1^ as the ecological management threshold: beyond this level of relative disturbance intensity, the tendency of grassland condition toward degradation became more pronounced, and ground‐surface exposure and the deterioration of community composition became the key factors limiting overall grassland condition. Managers should strengthen the monitoring of grassland ecological conditions and formulate appropriate management measures accordingly. Therefore, management decisions concerning small mammals should follow the precautionary principle and be coordinated with integrated grassland management measures, so as to maintain and enhance the sustainability of these alpine meadow ecosystems.

## Author Contributions

**Yifan Zhao:** data curation (equal), investigation (equal), methodology (equal), visualization (equal), writing – original draft (equal). **Jing Yang:** data curation (equal), investigation (equal), methodology (equal), supervision (equal). **Shien Ren:** data curation (equal), investigation (equal). **Dongni Guo:** data curation (equal), investigation (equal). **Jin Li:** data curation (equal), investigation (equal). **Shoushu Pang:** data curation (equal), investigation (equal). **Lufan Yang:** data curation (equal), investigation (equal). **Tongxi Zhang:** data curation (equal), investigation (equal). **Hongyu Wang:** data curation (equal), investigation (equal). **Xiaoning Nan:** conceptualization (equal), data curation (equal), project administration (equal), resources (equal), supervision (equal).

## Funding

This work was supported by the National Forestry and Grassland Administration (202401‐10‐5) and the National Key Research and Development Program of China (No. 2022YFD1400403‐05).

## Conflicts of Interest

The authors declare no conflicts of interest.

## Data Availability

The raw survey data are available at the following link: https://doi.org/10.6084/m9.figshare.32270151.
